# Empathic Cognitions Affected by Undetectable Social Chemosignals: An EEG Study on Visually Evoked Empathy for Pain in an Auditory and Chemosensory Context

**DOI:** 10.3389/fnbeh.2018.00243

**Published:** 2018-10-16

**Authors:** Matthias Hoenen, Katrin T. Lübke, Bettina M. Pause

**Affiliations:** Institute of Experimental Psychology, Heinrich Heine University Düsseldorf, Düsseldorf, Germany

**Keywords:** mu activity, empathy for pain, empathy, mirror neuron system, body odor, multimodal integration, audio-visual, social chemosignals

## Abstract

Reduction of mu activity within the EEG is an indicator of cognitive empathy and can be generated in response to visual depictions of others in pain. The current study tested whether this brain response can be modulated by an auditory and a chemosensory context. Participants observed pictures of painful and non-painful actions while pain associated and neutral exclamations were presented (Study 1, *N* = 30) or while chemosensory stimuli were presented via a constant flow olfactometer (Study 2, *N* = 22). Chemosensory stimuli were sampled on cotton pads while donors participated in a simulated job interview (stress condition) or cycled on a stationary bike (sport condition). Pure cotton was used as a control. The social chemosignals could not be detected as odors. Activity within the 8–13 Hz band at electrodes C3, C4 (mu activity) and electrodes O1, O2 (alpha-activity) was calculated using Fast-Fourier-Transformation (FFT). As expected, suppression of power in the 8–13 Hz band was stronger when painful as compared to non-painful actions were observed (Study 1, *p* = 0.020; Study 2, *p* = 0.005). In addition, as compared to the neutral auditory and chemosensory context, painful exclamations (Study 1, *p* = 0.039) and chemosensory stress signals (Study 2, *p* = 0.014) augmented mu-/alpha suppression also in response to non-painful pictures. The studies show that processing of social threat-related information is not dominated by visual information. Rather, cognitive appraisal related to empathy can be affected by painful exclamations and subthreshold chemosensory social information.

## Introduction

Affective empathy can be understood as the automatic activation of neural representations specific to the observed emotional state of others, resulting in the re-experience of someone else’s feelings (e.g., emotional contagion, Shamay-Tsoory et al., 2009; Perception-Action-Model of Empathy, Preston and de Waal, 2001). Observed movements and emotions of conspecifics are mirrored in brain areas involved in somatosensation and emotion processing (e.g., anterior insula and primary somatosensory cortex; Keysers and Gazzola, 2009; Keysers et al., 2010). Research on the neuronal mechanisms of empathy for pain is well established (for a review, see Lamm et al., 2011). Especially the primary somatosensory cortex (processing the sensory features of pain; Bushnell et al., 1999; Hofbauer et al., 2001) is activated during the observation of conspecifics in pain (for a review, see Keysers et al., 2010). Because mu activity (activity in the 8–13 Hz range within the human electroencephalogram) is inversely correlated to the activity of the somatosensory cortex (Pineda, 2005), it is a reliable indicator for the processes involved in empathy for pain: When painful actions are observed, mu activity is attenuated (mu suppression, e.g., Cheng et al., 2008; Yang et al., 2009; Chen et al., 2012). Cognitive appraisal (e.g., beliefs regarding the observed individual’s pain sensitivity) modulates mu suppression (Cheng et al., 2007; Perry et al., 2010a; Hoenen et al., 2015), matching the fact that mu suppression varies with cognitive empathic skills like perspective taking (Hoenen et al., 2013).

**Figure 1:**
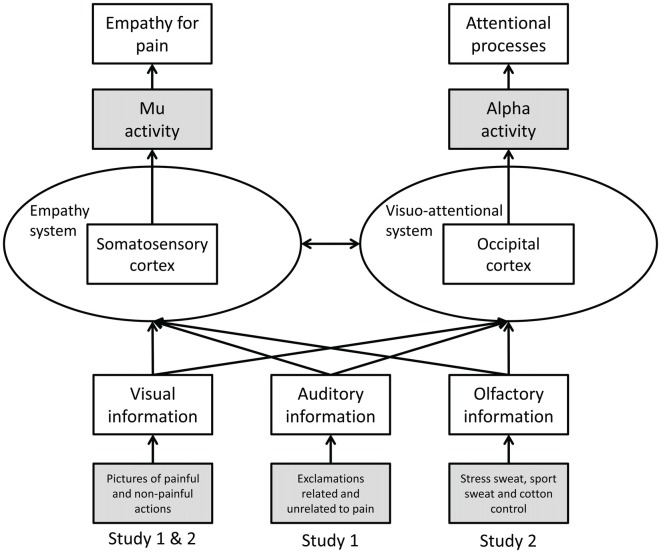
Schematic representation of Study 1 and 2. Operationalization of variables is marked in gray (dependent variables at the top, independent variables at the bottom).

To date most studies on empathy for pain have utilized unimodal stimuli, in particular, visual depictions of others in pain. However, in everyday life, social information is transmitted in a multimodal fashion, including visual, auditory and chemosensory cues (for a discussion of the ecological validity of neuroscientific research on empathy see Zaki and Ochsner, 2012). Whether in a multimodal social context a given modality (i.e., visual, auditory, or chemosensory) is processed preferentially, or whether the specific information (pain/no pain) is of importance regardless of the modality in which the information is presented, has not been investigated systematically in the context of empathy. Therefore, the current studies investigate visually induced empathy for pain in congruent and incongruent auditory (Study 1) and chemosensory (Study 2) contexts (see Figure 1).

## Study 1: Visually Induced Empathy for Pain in an Auditory Context

### Introduction

In general, visual action depicting stimuli induce more pronounced mu suppression than auditory stimuli (Kaplan and Iacoboni, 2007; McGarry et al., 2012), indicating that mu suppression prominently relies on visual information (McGarry et al., 2012). However, the human mirror neuron system integrates multimodal information about perceived actions: cortico-motor-excitability of the primary motor cortex increases in response to congruent audio-visual action depicting stimuli as compared to incongruent audio-visual action depicting stimuli (Alaerts et al., 2009), and audio-visual compared to unimodal action related stimuli facilitate mirror neuron system activity (McGarry et al., 2012). To our knowledge, only one study investigated the effects of auditory stimuli on empathy for pain, showing that pain related screams (as compared to laughing or snoring) elicit activity in empathy related neural structures (left insula, secondary somatosensory cortices; Lang et al., 2011).

In the current study, visual stimuli (depictions of painful and non-painful actions) and auditory stimuli (exclamations related and unrelated to pain) were presented either congruently or incongruently. If the specific information *pain* is processed preferentially, stronger mu suppression should be evident in response to any pain related information (whether presented congruently or incongruently) as compared to congruent non-painful information. If the visual modality is processed preferentially, visual pain-related information should induce stronger mu suppression than visual non-painful information, regardless of the information presented auditorily. Mu activity was recorded above central electrode positions. Additionally, occipital alpha activity was recorded in order to ensure that mu responses were specific for pain empathy and not merely reflecting visual and attentional processes (Sauseng et al., 2005; Hoenen et al., 2015).

### Materials and Methods

#### Participants

A total of 30 right-handed (assessed using Annett, 1967) volunteers (18 females) participated in the experiment. All participants reported to be healthy and free of neurological or psychiatric conditions. Participants had a mean age of 23.5 years (*SD* = 3.8, range: 19.5–37.7). Participants gave their written informed consent and were compensated with course credit or €10. The experiment was approved by the ethics committee of the Faculty of Mathematics and Natural Sciences of the Heinrich-Heine-University Düsseldorf.

#### Material

A set of 64 color pictures was used, showing common actions with painful or non-painful outcome (56 depictions of right hands and eight depictions of right feet; resolution: 600 × 450 pixel). The 32 painful actions corresponded to 32 non-painful actions (e.g., cutting a cucumber, while a finger is or is not placed between knife and cucumber). Various types of pain (mechanical, thermal, and pressure) were represented. The pictures were selected from a set of 128 pictures which has been validated in several studies (Jackson et al., 2005; Cheng et al., 2008; Yang et al., 2009; Hoenen et al., 2015). The pictures were shown (Presentation 14, Neurobehavioral Systems Inc., Berkeley, CA, USA) on a TFT monitor (resolution: 1280 × 1024 pixel; model: Terra LCD 4319; Wortmann AG, Germany) at a distance of 75 cm to the participant’s eyes, covering a visual angle of 13.4° horizontal and 10.1° vertical. A white cross (2.2° × 2.2° visual angle) on a black background served to record baseline activity.

A set of 64 audio recordings of exclamations (e.g., “ah!,” or “oh!”) intonated either in a painful (32) or neutral fashion (32) served as auditory stimuli. The duration of the auditory stimuli (*M* = 1.08 s, *SD* = 0.26) did not differ between these two conditions (*t*_(62)_ = 0.66, *p* = 0.513). In a preliminary study (*N* = 9), the auditory pain stimuli were rated as more painful (*M* = 66.83, *SD* = 8.32) than the neutral auditory stimuli (*M* = 4.56, *SD* = 4.01; *t*_(62)_ = 38.13, *p* < 0.001) via computer-based visual analog scales (length 18.5 cm; 0 = *not painful*, 100 = *very painful*).

Participants completed the *Saarbrueck Personality Questionnaire on Empathy* (SPQ; Paulus, 2009), a German adaptation of the Interpersonal Reactivity Index (IRI; Davis, 1983), a self-report of empathic abilities. The SPQ assesses the empathic dimensions fantasy (tendency to transpose oneself imaginatively in the feelings of fictitious characters), perspective taking (adopting the psychological view of others), empathic concern (sympathy and concern for unfortunate others), and personal distress (feeling of unease in tense interpersonal settings; Davis, 1983). Each dimension is reflected by a subscale consisting of four items, ranging from 4 (low empathy) to 20 (high empathy).

#### Procedure

**Figure 2:**
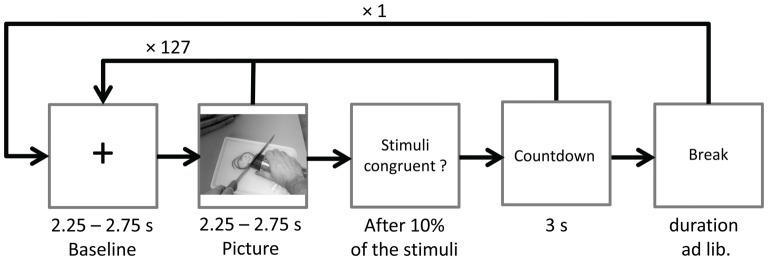
Time course of Study 1. The sound (congruent or incongruent) started with picture onset and had a mean duration of 1.08 s (*SD* = 0.26).

Ongoing EEG was recorded during two identical blocks with 128 stimulus pairs each. After the first block the word *break* appeared on screen, giving participants time to relax and to move their eyes. Participants could start the next block themselves by mouse-click. To ensure suppression of eye-blinks during EEG recordings, the instruction *Please don’t blink in* followed by a countdown from three to one (duration 3 s) was presented prior to the start of each block, and also after the ratings (see below). Pictures were presented in randomized order for a random duration of 2.25–2.75 s. In between picture presentations a fixation cross (baseline) was presented for a random duration varying between 2.25 s and 2.75 s. Each picture was paired with either a randomly chosen congruent or incongruent sound starting with the onset of picture presentation (see Figure 2).

In 10% of the trials participants were asked to indicate via mouse click whether a congruent or incongruent stimulus pair had been presented. At the end of the session, participants completed the SPQ.

#### EEG Recording and Analysis

EEG was recorded from 16 Ag/AgCl sintered active electrodes (positions Fp1, Fp2, F3, Fz, F4, C3, Cz, C4, P3, Pz, P4, O1, Oz and O2 of the 10/10-system and earlobes), embedded in a stretch lycra cap (actiCAP, 32-channel standard 2 layout, Brain Products GmbH, Germany). Eye movements were monitored using a bipolar montage with Fp2 acting as the supraorbital electrode, and the suborbital electrode displaced 1 cm lateral from the vertical axis of the eye. The ground electrode was placed at position AFz. Data were sampled at 500 Hz (bandwidth: DC—135 Hz) with the left earlobe as reference, using a V-Amp EEG System (Brain Products GmbH, Germany).

During offline processing, data were re-referenced to averaged earlobes and filtered with a high-pass filter at 0.5 Hz (48 dB/oct), a low-pass filter at 40 Hz (48 dB/oct), and a notch filter at 50 Hz (BrainVision Analyzer 2, Brain Products GmbH, Germany). Eye movements were corrected using an independent component analysis including data from all electrodes (Jung et al., 1998).

In order to quantify the power within the mu range (8–13 Hz), the EEG was segmented into epochs of 512 data points (1024 ms), beginning 100 data points (200 ms) after the onset of the fixation-cross (baseline) or the picture (experimental data). The 200 ms delay was introduced in order to reduce effects of early event-related potentials on the EEG (for similar approaches see Whitmarsh et al., 2011; Hoenen et al., 2015). The epochs were visually inspected for remaining artifacts (e.g., movement artifacts), and a total of 5.7% of data were rejected. For the frequency-analysis, a non-complex Fast-Fourier-Transform (FFT; Hanning-Windowing with *α* = 0.50; frequency resolution of 0.977 Hz) was applied to each epoch.

For each frequency bin of the experimental FFT data, the attenuation relative to baseline (suppression index) was calculated in dB (10× log transformed ratio of the power during the experimental condition (picture) relative to the power during baseline (fixation cross)).

In accordance with previous studies, electrodes C3 and C4 were used to measure mu activity, while O1 and O2 served as a control for alpha-activity (e.g., Oberman et al., 2007; Cheng et al., 2008; Perry et al., 2010b; Hoenen et al., 2013).

#### Statistical Analysis

In order to test whether the effects of the depicted actions on the power in the mu range are modulated by sound stimuli, a repeated-measures ANOVA was conducted, comparing the suppression indices across PICTURE (painful, non-painful) SOUND (painful, non-painful), LATERALITY (left, right) and REGION (central, occipital) as within-subject factors. Nested effects were calculated in accordance with Braver et al. (2003).

Furthermore, in order to test whether participants’ dispositional empathy corresponds to mu suppression in response to observed painful actions in congruent and incongruent auditory context, the differences of suppression indices between the PICTURE conditions (painful minus non-painful) in both SOUND conditions were correlated with the SPQ scores (Pearson’s product-moment correlation; collapsed across lateral electrode positions).

In order to verify that the stimulus pairs elicit suppression of mu- and alpha activity relative to baseline, one sided *t*-tests against zero (bonferroni-corrected) of the suppression-indices of each electrode in each condition (congruent painful, congruent non-painful, painful picture with non-painful sound, non-painful picture with painful sound) were conducted. All analyses were conducted using SPSS 24 (IBM Corp., Armonk, NY, USA).

### Results

#### Mu-/Alpha Suppression

Regardless of the condition, suppression indices were smaller than zero at each electrode position, indicating that the perception of human actions in an auditory context is related to suppression in the 8–13 Hz band relative to baseline (all *p*s < 0.001).

#### Modulation of Mu-/Alpha Activity by Visual and Auditory Stimuli

**Figure 3:**
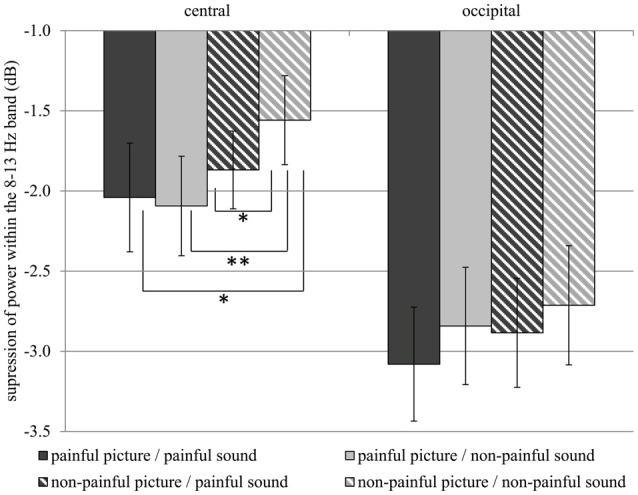
Study 1. Suppression within the 8–13 Hz band at central (C3, C4) and occipital (O1, O2) electrode sites. Suppression at central electrode sites is always stronger when a stimulus containing pain information is present as compared to a non-painful picture paired with non-painful sound (Interaction PICTURE × SOUND × REGION: *F*_(1,29)_ = 6.34, *p* = 0.018, $\eta_{\text{p}}^{2}$ = 0.179). The error bars represent the standard error. *p < 0.05, **p < 0.01.

Table 1 gives an overview of all effects including the factors PICTURE or SOUND. In general, suppression within the 8–13 Hz band was stronger when painful (*M* = −2.51, *SD* = 1.52) as compared to non-painful actions (*M* = −2.26, *SD* = 1.39) were observed (main effect PICTURE: *F*_(1,29)_ = 6.02, *p* = 0.020, $\eta_{\text{p}}^{2}$ = 0.172). Further analysis showed that, at central electrode sites, intensified mu suppression in response to painful actions (*M* = −2.09, *SD* = 1.70) compared to non-painful actions (*M* = −1.56, *SD* = 1.52) was especially evident when pictures were paired with non-painful sounds (PICTURE × SOUND × REGION: *F*_(1,29)_ = 6.34, *p* = 0.018, $\eta_{\text{p}}^{2}$ = 0.179; PICTURE × SOUND in central: *F*_(1,29)_ = 4.48, *p* = 0.043, $\eta_{\text{p}}^{2}$ = 0.134; PICTURE in auditory non-painful in central: *F*_(1,29)_ = 12.06, *p* = 0.002, $\eta_{\text{p}}^{2}$ = 0.294; see Figure 3). In contrast, in the context of painful sounds, mu activity did not differentiate between painful and non-painful actions (PICTURE in auditory painful in central: *F*_(1,29)_ = 1.00, *p* = 0.325, $\eta_{\text{p}}^{2}$ = 0.033). When focusing on the effects of painful vs. non-painful sounds directly, results showed enhanced mu suppression in response to non-painful actions in the painful auditory context (*M* = −1.87, *SD* = 1.33) compared to the non-painful auditory context, again specifically for central electrode sites (SOUND in visual non-painful in central: *F*_(1,29)_ = 4.72, *p* = 0.039, $\eta_{\text{p}}^{2}$ = 0.149; see Figure 3). In contrast, when a painful action was presented, mu activity did not differentiate between painful and non-painful sounds (SOUND in visual painful in central: *F*_(1,29)_ = 0.11, *p* = 0.743, $\eta_{\text{p}}^{2}$ = 0.004). At occipital electrode sites, no interaction between picture and sound was evident (PICTURE × SOUND in occipital:* F*_(1,29)_ = 0.51, *p* = 0.482, $\eta_{\text{p}}^{2}$ = 0.018).

**Table 1:** Study 1: main effects, interactions and simple effects including the factors picture or sound.

Main effect/interaction	Simple effects	2nd order simple effects	Single comparisons	2nd order simple effects	Single comparisons
Picture: *F*_(1,29)_ = 6.02, *p* = 0.020, $\eta_{\text{p}}^{2}$ = 0.172	-	-	Pain < No-Pain*	-	-
Picture × Laterality × Region: *F*_(1,27)_ = 5.49, *p* = 0.026, $\eta_{\text{p}}^{2}$ = 0.159	Picture × Laterality in Central: *F*_(1,29)_ = 6.58, *p* = 0.016, $\eta_{\text{p}}^{2}$ = 0.185	Picture in Left in Central: *F*_(1,29)_ = 10.01, *p* = 0.004, $\eta_{\text{p}}^{2}$ = 0.256	Visual Pain < Visual No-Pain**	-	-
		Picture in Right in Central: *F*_(1,29)_ = 2.75, *p* = 0.108, $\eta_{\text{p}}^{2}$ = 0.087	-	-	-
	Picture × Laterality in Occipital: *F*_(1,29)_ < 0.01, *p* = 0.958, $\eta_{\text{p}}^{2}$ < 0.001	-	-		-
Picture × Sound × Region: *F*_(1,29)_ = 6.34, *p* = 0.018, $\eta_{\text{p}}^{2}$ = 0.179	Picture × Sound in Central: *F*_(1,29)_ = 4.48, *p* = 0.043, $\eta_{\text{p}}^{2}$ = 0.134;	Picture in Auditory Pain in Central: *F*_(1,29)_ = 1.00, *p* = 0.325, $\eta_{\text{p}}^{2}$ = 0.033	-	Sound in Visual No-Pain in Central: *F*_(1,29)_ = 4.72, *p* = 0.039, $\eta_{\text{p}}^{2}$ = 0.149	Auditory Pain < Auditory No-Pain**
		Picture in Auditory No-Pain in Central: *F*_(1,29)_ = 12.06, *p* = 0.002, $\eta_{\text{p}}^{2}$ = 0.294	Visual Pain < Visual No-Pain**	Sound in Visual Pain in Central: *F*_(1,29)_ = 0.11, *p* = 0.743, $\eta_{\text{p}}^{2}$ = 0.004	-
	Picture × Sound in Occipital:* F*_(1,29)_ = 0.51, *p* = 0.482, $\eta_{\text{p}}^{2}$ = 0.018	-	-	-	-

*p < 0.05. **p < 0.005.

In general, suppression within the 8–13 Hz range was stronger at occipital (*M* = −2.88, *SD* = 1.92) as compared to central electrode sites (*M* = −1.89, *SD* = 1.51; main effect REGION: *F*_(1,29)_ = 7.74, *p* = 0.009, $\eta_{\text{p}}^{2}$ = 0.211), and stronger at left electrode sites (*M* = −2.52, *SD* = 1.38) as compared to right electrode sites (*M* = −2.25, *SD* = 1.52; main effect LATERALITY: *F*_(1,29)_ = 6.31, *p* 0.018, $\eta_{\text{p}}^{2}$ = 0.179). Moreover, above the central scalp region the observation of painful actions elicited stronger suppression at the left (C3; *M* = −2.27, *SD* = 1.76) compared to the right electrode site (C4; *M* = −1.80, *SD* = 1.40; PICTURE × LATERALITY × REGION: *F*_(1,27)_ = 5.49, *p* = 0.026, $\eta_{\text{p}}^{2}$ = 0.159; PICTURE × LATERALITY in central: *F*_(1,29)_ = 6.58, *p* = 0.016, $\eta_{\text{p}}^{2}$ = 0.185; PICTURE in left in central: *F*_(1,29)_ = 10.01, *p* = 0.004, $\eta_{\text{p}}^{2}$ = 0.256). At occipital electrode sites no lateralization of the PICTURE effect was observed (PICTURE × LATERALITY in occipital: *F*_(1,29)_ < 0.01, *p* = 0.958, $\eta_{\text{p}}^{2}$ < 0.001).

#### Correlation of SPQ Scores With Differences of Suppression Indices Between Picture Conditions in Both Sound Conditions

**Figure 4:**
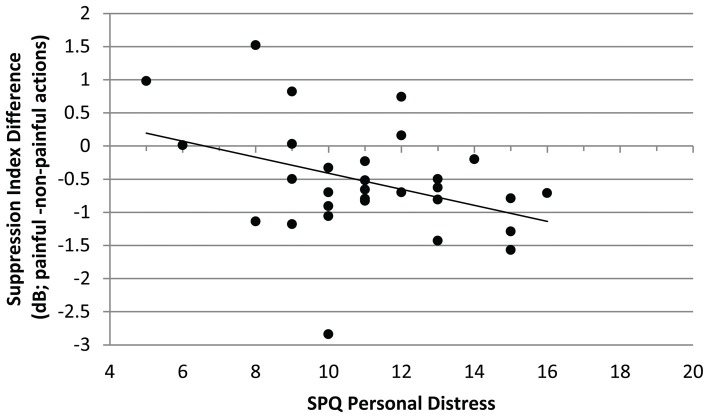
Study 1. Correlation of mu-suppression difference for painful minus non-painful actions in the non-painful sound context with the personal distress self-ratings (Saarbrueck Personality Questionnaire on Empathy (SPQ); *r* = −0.378, *p* = 0.040). The higher participants scored on the scale, the stronger the mu suppression in response to the observation of painful relative to non-painful actions.

The mu-suppression difference of painful minus non-painful actions in the non-painful sound context (at central electrode sites) was negatively associated with participants’ self-reported degree of personal distress (*r* = −0.378, *p* = 0.040; see Figure 4). Although the correlation is negative, it represents a positive relationship between mu suppression differences and personal distress, because larger differences in mu suppression are represented by more negative numbers.

No other correlation was significant.

### Discussion

The results show that mu suppression is enhanced whenever a stimulus containing pain related information is perceived. This effect does not vary with the specific modality (auditory or visual) containing the pain related information. The data do not support beneficial effects of congruency, as mu suppression in response to congruent visual-auditory pain related information does not exceed mu suppression in response to pain related information, which is present in only one modality. Furthermore, the results show that the higher the participants’ self-reported degree of personal distress, the more sensitive these participants were to visual pain-related information in a neutral auditory context. Thus, individuals stressed by others’ pain seem to exhibit more effective mirroring processes. In line with the current findings, observing others in pain elicits affective distress in the observer (Craig, 1968).

In total, the effects of pain perception on mu activity are specific for central electrode sites, as at occipital electrode sites no interaction between visual and auditory information was evident. The results indicate that the auditory and visual perception of pain affect mu activity within the human mirror neuron system (Arnstein et al., 2011), but does not affect nonspecific attentional processes, which are associated with changes in occipital alpha activity (Sauseng et al., 2005; Woodruff et al., 2011).

The current study shows that the mirror neuron system is tuned to socially significant stimuli, containing survival (pain) related information, independent of the sensory modality (visual, auditory). It also confirms the results of Lang et al. (2011), showing that the human mirror neuron system can be activated by auditory pain related stimuli. Thus, regardless of the modality, the mirror neuron system seems to represent the meaning of action outcomes (Galati et al., 2008; Hoenen et al., 2015).

In comparison to social signals of other modalities, social chemosignals bear unique features (Pause, 2017): they cannot be intentionally manipulated by the signal sender and are therefore considered to be *honest signals*. In addition, they are effective irrespective of conscious detection. It has thus been speculated that chemosensory signals have a processing advantage relative to social signals of other modalities (e.g., Pause et al., 2004; Adolph and Pause, 2012). Therefore, the current study was rerun using social chemosignals containing threat-related information instead of auditory stimuli.

## Study 2: Visually Induced Empathy for Pain in a Chemosensory Context

### Introduction

Social communication (including emotional contagion) is one of the main functions of human chemosensation (Stevenson, 2010; Pause, 2012). Stress-related chemosignals adjust social perception towards potential threat by diminishing visual acuity for social safety signals (Pause et al., 2004; Zernecke et al., 2011) and enhancing visual acuity for social signals related to harm (Mujica-Parodi et al., 2009; Zhou and Chen, 2009). Furthermore, motor systems related to withdrawal behavior (startle response) are automatically primed by stress-related chemosignals (Prehn et al., 2006; Pause et al., 2009).

Barely detectable anxiety sweat samples activate brain areas involved in the processing of social emotional stimuli (fusiform gyrus), and in the regulation of empathic feelings (insula, precuneus, cingulate cortex; Prehn-Kristensen et al., 2009). Moreover, anxiety related sweat is as potent as audiovisual fear signals in eliciting fear associated facial expressions, even when incongruent audiovisual information is present (de Groot et al., 2014). Together, these results indicate emotional contagion induced by chemosensory stress signals (Pause, 2017).

To our knowledge, so far only one study investigated the effects of olfactory stimuli on visual action perception, showing strongest activity of the mirror neuron system when the action target object (e.g., an apple) was both seen and smelled (Tubaldi et al., 2011). However, in the present study, social perception was assessed rather than olfactory perception and therefore social chemosignals were presented instead of common odors. As social chemosignals are not processed by olfactory, but by social brain areas, the processing of social chemosignals seems not to be comparable to the processing of common odors (Pause, 2012). Therefore, this study is the first to show how social chemosignals affect empathy related pain perception. In the current study, visual stimuli (painful and non-painful actions) and chemosensory stimuli (stress sweat/emotionally neutral sweat) were combined either in a congruent or an incongruent manner. The hypotheses are similar to the first study: if specific information (pain/stress) is processed preferentially, stronger mu suppression should be evident when pain related information is present in at least one modality, as compared to congruent non-painful information. If a specific modality (visual or chemosensory) is processed preferentially, pain-related information in this modality should induce stronger suppression of mu activity than non-painful information, regardless of the information in the other modality. Again, occipital alpha activity, reflecting visual and attentional processes, served as a control for the centrally dominant mu activity (Sauseng et al., 2005; Hoenen et al., 2015).

### Materials and Methods

#### Participants

A total of 29 right-handed (assessed using Annett, 1967) volunteers participated in the experiment. All participants reported to be non-smokers, healthy, free of neurological or psychiatric conditions, and not to suffer from diseases of the upper respiratory tract. Due to the suspicion of general hyposmia (*n* = 1), technical problems of the olfactometer (*n* = 3), and data loss due to a mismatch of breathing cycle and stimulus onset (*n* = 3), seven participants were excluded. The final sample consisted of 11 females and 11 males (*N* = 22) with a mean age of 23.8 years (*SD* = 4.7; range: 18.7–41.3). Participants gave their written informed consent and were compensated with course credit or €20. The experiment was approved by the ethics committee of the Faculty of Mathematics and Natural Sciences of the Heinrich-Heine-University Düsseldorf.

#### Material

##### Visual Stimuli

The same set of color pictures as in Study 1 was used, and pictures were presented the same way. Again, a white cross on a black background served to record baseline activity.

##### Chemosensory Stimuli

Axillary sweat was sampled from 16 men. Donors were on average 26.4 years old (*SD* = 6.8, range = 20.0–44.0). As axillary sweat production is in part genetically determined, and the respective allelic profiles vary with ethnos (Martin et al., 2010), only sweat donors of European origin were included. Donors were free of any neurological, psychiatric, endocrine, or immunological conditions, and reported not to use any acute or chronic medication. In addition, none of the donors reported using drugs or smoking cigarettes. Their body-mass-index ranged from 20.8 kg/m^2^ to 28.9 kg/m^2^ (*M* = 23.5, *SD* = 1.9). All donors gave written informed consent, and were paid for their donation. None of the sweat donors acted as a participant within the current study.

The donors were instructed to refrain from eating garlic, onions, asparagus, or any other spicy or aromatic food during the 24 h prior to the odor donation. Donors were also asked to refrain from using deodorants within this timeframe, and to wash their armpits exclusively with an odorless medical washing lotion (Eubos^®^, Dr. Holbein GmbH, Germany).

For collecting the axillary sweat, one cotton pad was fixed in each of the donor’s armpits. The axillary sweat was sampled for 90 min during a modified trier social stress test (TSST; Kirschbaum et al., 1993 stress condition) and a sport control condition (sport control). The TSST started with a first anticipatory phase (duration: 20 min), in which participants prepared three controversial topics (animal experiments, death penalty, and personal strengths and weaknesses) for a 5 min oral presentation. Subsequently, the participants gave an oral presentation on one of these topics and performed a mental arithmetic task (serially subtracting 17 from 2,043 as quickly and accurately as possible for 5 min; if a mistake was made, the experimenter interrupted, by stating “Incorrect. Start again.”). In order to enhance the feeling of social evaluative threat, both tasks were performed in front of a reserved female and male experimenter (introduced as experts in the evaluation of social behavior), participants were told that they were videotaped (no actual recording took place), and that their verbal and non-verbal behavior would be scored. Afterwards, participants evaluated common odors for 20 min (as part of the study described in Hoenen et al., 2017). The participants were then instructed that they had to prepare a difficult philosophical text for an oral exam 20 min later. However, the oral examination was not carried out and the session ended after this second anticipatory phase. The sport control sweat was obtained from the participants during cycling on a stationary bicycle. For each participant the training load was adjusted to result in a heart rate comparable to the mean heart rate measured during the TSST session, ensuring comparable physiological arousal (for further details on the sweat sampling procedure see Hoenen et al., 2017).

Sweat donors reported higher levels of anxiety and anger in the stress condition than in the sport control condition (anxiety, main effect session, *F*_(1,17)_ = 24.55, *p* < 0.001; anger increased during the stress session, interaction session x time: *F*_(2,36)_ = 4.51, *p* = 018). Furthermore, participants showed a stronger increase of cortisol (relative to the beginning of the session) during the stress condition than during the sport control condition (interaction session × time: *F*_(2,36)_ = 13.40, *p* < 0.001).

Following the completion of collection, all cotton pads were chopped and pooled with respect to the donation condition. The pooled samples were divided into portions of 0.3 g and stored at −20°C. A sample of pure cotton pads, serving as a second control condition (cotton control), was treated exactly the same.

Chemosensory stimuli were presented using a constant-flow six channel olfactometer (OM6b, Burghart, Germany; flow rate: 100 ml/s; stimulus duration: 3,000 ms; inter-stimulus-interval: 10,000 ms). Both nostrils were stimulated simultaneously, and both air streams were controlled by separate mass flow meters. The temperature of the air flow at the exit of the olfactometer was 37°C and the relative humidity was set above 80%. Pink noise of 80 dB(A) was presented binaurally over earplugs (Etymotic Research, ER3–14A), in order to prevent the participants from hearing the switching valves of the olfactometer.

##### Olfactory Hyposmia Screening

All participants were briefly screened for general hyposmia: participants were asked to discriminate a bottle containing phenyl-ethyl alcohol (99%, Fluka, Germany, 1:100 [v/v] diluted in diethyl phthalate) from a set of three bottles in at least two out of three trials, with the two distractor bottles containing the same volume of solvent (three alternative forced choice). Phenyl-ethyl alcohol was chosen as test odorant for general hyposmia since it is considered a purely olfactory odor used as a standard in olfactory sensitivity testing (Doty, 1997) and to date no case of specific anosmia to phenyl-ethyl alcohol has been reported (Croy et al., 2015). The brief screening test revealed suspicion of general hyposmia in one participant who consequently was excluded from data analysis.

##### Odor Detection

To determine participants’ ability to detect an odor from the sweat samples, the participants were asked to discriminate the target odor (either stress sweat or sport sweat) from two distractors (pure unused cotton; three alternative forced choice). The targets and distractors were presented in a random sequence via the olfactometer (stimulus duration = 5 s; interstimulus interval = 5 s). The task was repeated five times.

##### Ratings

In order to investigate the olfactory features of the sweat samples, the participants evaluated the chemosensory profile of the sweat samples regarding intensity, pleasantness, unpleasantness, and familiarity using pictographic computerized nine level likert-scales (range: 1–9). Again, the chemosensory stimuli were presented via the olfactometer (stimulus duration = 5 s for each scale).

The action depicted in each of the pictures was rated for the degree of painfulness on computer-based visual analog scales (length 18.5 cm; 0 = *not painful*, 100 = *very painful*). Additionally, participants completed the *Saarbrueck Personality Questionnaire on Empathy* (see “Study 1: Visually Induced Empathy for Pain in An Auditory Context” section).

#### Procedure

Prior to the EEG-recording, the chemosensory profile of the sweat samples was assessed and the odor detection test was carried out.

The EEG session consisted of three blocks with 64 stimuli pairs each. Between blocks, the word *break* appeared, giving participants some time to relax and to move their eyes. Participants could start the next block themselves by mouse-click. The participants were instructed to avoid eye movements and blinks during the presentation of both the fixation cross and the picture. In total, each picture was presented three times, and each time it was paired with another chemosensory context odor (stress condition, sport control, cotton control). Pictures were presented in pseudo-randomized order. The same picture was never presented twice in succession.

**Figure 5:**
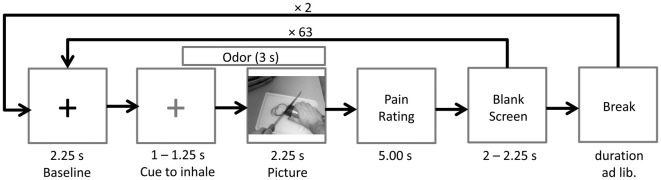
Time course of Study 2. The odor (cotton control, sport control or stress condition) was presented 0.75 s prior to picture onset. A blue cross (here depicted in gray), instead of the black cross (baseline), signaled the participants to inhale until picture offset.

Prior to picture onset a fixation cross (baseline) was presented for a random duration varying between 3,250 ms and 3,500 ms. After 2,250 ms the fixation cross changed its color from white to blue, indicating that the participant had to inhale until picture offset. Following the fixation cross, the picture was presented for a random duration varying between 2,250 ms and 2,750 ms. The chemosensory stimulus was presented for 3000 ms, starting 750 ms prior to picture onset. After each picture, participants were asked to rate the painfulness of the observed action on a visual analog scale (fixed duration 5,000 ms). Following a blank screen (duration 2,000–2,250 ms), the next baseline was presented (see Figure 5). At the end of the session, participants completed the SPQ.

#### EEG Recording and Analysis

In Study 2, the same apparatus as in Study 1 was used for EEG recordings. In addition, participants’ breathing cycles were recorded with one respiration belt (Brain Products, Germany) attached to the abdomen and one respiration belt attached to the thorax.

The same data reduction procedure as in Study 1 was applied to the data of Study 2. In addition to the artifact rejection, segments were rejected if the participant did not inhale constantly during an interval from 500 ms prior to picture onset to 500 ms after picture onset. In total 5.8% of the data were rejected. For the FFT a time-window from 200 ms to 2,248 ms (duration 2,048 ms) after stimulus onset was used, resulting in a frequency resolution of 0.488 Hz.

#### Statistical Analysis

A repeated-measures ANOVA was conducted to compare the suppression indices (attenuation relative to baseline in dB, see “Study 1: EEG Recording and Analysis” section) across PICTURE (painful, non-painful), CHEMOSENSORY CONTEXT (stress condition, sport control, cotton control), LATERALITY (left, right) and REGION (central, occipital) as within-subject factors. Nested effects were calculated in accordance with Braver et al. (2003).

Furthermore, the differences of suppression indices between the PICTURE conditions (painful minus non-painful) in all three CHEMOSENSORY CONTEXT conditions were correlated with the SPQ scores (Pearson’s product-moment correlation; collapsed across all electrode positions).

Suppression relative to baseline was tested using one sided *t*-tests against zero (bonferroni-corrected) for the suppression-indices of each electrode in each condition.

In order to determine whether the chemosensory context affects the perceived painfulness of the actions, pain ratings were subjected to a repeated-measures ANOVA with the factors PICTURE (painful, non-painful) and CHEMOSENSORY CONTEXT (stress condition, sport control, cotton control).

All analyses were conducted using SPSS 24 (IBM Corp., Armonk, NY, USA). Degrees of freedom were corrected using Greenhouse-Geisser ε whenever necessary.

### Results

#### Mu-/Alpha Suppression

Suppression relative to baseline was smaller than zero in all conditions at each electrode site, indicating that the perception of human actions in a chemosensory context is related to suppression in the 8–13 Hz band (all *p*s < 0.005, except suppression at electrode O1 in the non-painful/cotton control condition, *p* = 0.013).

#### Modulation of Mu-/Alpha-Activity by Visual and Chemosensory Stimuli

**Figure 6:**
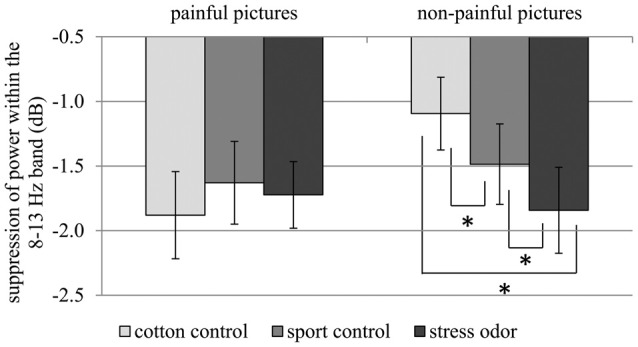
Study 2. Suppression within the 8–13 Hz band collapsed across central and occipital electrode sites. The chemosensory context had an effect on suppression when non-painful pictures were observed, but not when painful pictures were observed (Interaction CHEMOSENSORY CONTEXT × PICTURE: *F*_(2,42)_ = 5.12, *p* = 0.010, $\eta_{\text{p}}^{2}$ = 0.196). The error bars represent the standard error. *p < 0.05.

In response to painful compared to non-painful actions, pronounced suppression of activity within the 8–13 Hz range was only evident in the cotton control context (CHEMOSENSORY CONTEXT × PICTURE: *F*_(2,42)_ = 5.12, *p* = 0.010, $\eta_{\text{p}}^{2}$ = 0.196; PICTURE within cotton control: *F*_(1,21)_ = 9.96, *p* = 0.005, $\eta_{\text{p}}^{2}$ = 0.322; see Figure 6). Chemosensory stress signals especially affected suppression within the 8–13 Hz band when non-painful actions were observed. In detail, suppression was stronger in response to non-painful actions presented in the chemosensory stress context (*M* = −1.84, *SD* = 1.56) compared to both the sport control context (*M* = −1.49, *SD* = 1.46;* t*_(21)_ = 2.10, *p* = 0.048) and cotton control context (*M* = −1.09, *SD* = 1.32;* t*_(21)_ = 2.68, *p* = 0.014; CHEMOSENSORY CONTEXT in non-painful: *F*_(2,42)_ = 6.38, *p* = 0.013, $\eta_{\text{p}}^{2}$ = 0.233; see Figure 6). Furthermore, during the presentation of non-painful actions, suppression was stronger in the sport control context than in the cotton control context (*t*_(21)_ = 2.47, *p* = 0.022). In contrast, when painful actions were presented, no effects of the chemosensory context were observed (CHEMOSENSORY CONTEXT in painful, *F*_(2,42)_ = 0.86, *p* = 0.429, $\eta_{\text{p}}^{2}$ = 0.040).

Due to the disordinal interaction CHEMOSENSORY CONTEXT × PICTURE, the main effect of PICTURE (*F*_(1,21)_ = 8,91, *p* = 0.007, $\eta_{\text{p}}^{2}$ = 0.298), and the interactions of REGION × PICTURE (*F*_(2,42)_ = 4.78, *p* = 0.040, $\eta_{\text{p}}^{2}$ = 0.185) and REGION × CHEMOSENSORY CONTEXT (*F*_(2,42)_ = 3.59, *p* = 0.036, $\eta_{\text{p}}^{2}$ = 0.146) could not be interpreted (see Table 2).

**Table 2:** Study 2: main effects, interactions and simple effects including the factors picture or chemosensory context.

Main effect/interaction	Simple effects	Single comparisons	Simple effects	Single comparisons
Picture: *F*_(1,21)_ = 8,91, *p* = 0.007, $\eta_{\text{p}}^{2}$ = 0.298	-	Pain < No-Pain**	-	-
Chem. Context × Picture: *F*_(2,42)_ = 5.12, *p* = 0.010, $\eta_{\text{p}}^{2}$ = 0.196	Chem. Context in No-Pain: *F*_(2,42)_ = 6.38, *p* = 0.013, $\eta_{\text{p}}^{2}$ = 0.233	Cotton < Sport* Cotton < Stress* Sport < Stress*	Picture in Cotton: *F*_(1,21)_ = 9.96, *p* = 0.005, $\eta_{\text{p}}^{2}$ = 0.322	Pain < No-Pain**
	Chem. Context in Pain, *F*_(2,42)_ = 0.86, *p* = 0.429, $\eta_{\text{p}}^{2}$ = 0.040	-	Picture in Sport: *F*_(1,21)_ = 0.61, *p* = 0.445, $\eta_{\text{p}}^{2}$ = 0.028	-
			Picture in Stress: *F*_(1,21)_ = 1.06, *p* = 0.316, $\eta_{\text{p}}^{2}$ = 0.048	-
Picture × Region: *F*_(2,42)_ = 4.78, *p* = 0.040, $\eta_{\text{p}}^{2}$ = 0.185	Picture in Central, *F*_(2,42)_ = 2.28, *p* = 0.146, $\eta_{\text{p}}^{2}$ = 0.098	Pain < No-Pain**	-	-
	Picture in Occipital, *F*_(2,42)_ = 14.20, *p* = 0.001, $\eta_{\text{p}}^{2}$ = 0.403	-	-	-
Chem. Context × Region *F*_(2,42)_ = 3.59, *p* = 0.036, $\eta_{\text{p}}^{2}$ = 0.146	Chem. Context in Central, *F*_(2,42)_ = 1.31, *p* = 0.281, $\eta_{\text{p}}^{2}$ = 0.059	Stress < Sport * Stress < Cotton*	-	-
	Chem. Context in Occipital, *F*_(2,42)_ = 4.42, *p* = 0.018, $\eta_{\text{p}}^{2}$ = 0.174	-	-	-

*p < 0.05. **p < 0.005.

#### Correlation of SPQ Scores With Differences of Suppression Indices Between Picture Conditions Within all Chemosensory Contexts

**Figure 7:**
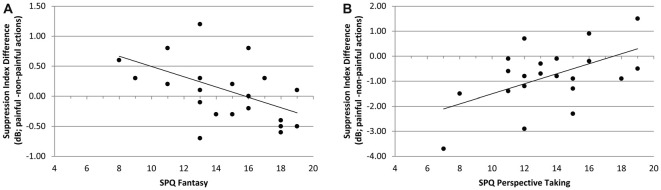
Study 2. **(A)** Correlation of mu-suppression difference for painful minus non-painful actions in chemosensory stress context with the fantasy self-ratings (SPQ; *r* = −0.490, *p* = 0.021). The higher participants scored on the fantasy scale, the stronger the mu suppression in response to the observation of painful relative to non-painful actions. **(B)** Correlation of mu-suppression difference for painful minus non-painful actions in the chemosensory stress context with the perspective taking self-ratings (*r* = 0.531, *p* = 0.011). The higher participants scored on the perspective taking scale, the weaker the mu suppression in response to the observation of painful relative to non-painful actions.

In the context of chemosensory stress signals, the mu-suppression difference of painful minus non-painful actions was negatively associated with participants’ scores on the SPQ’s fantasy scale (*r* = −0.490, *p* = 0.021; see Figure 7A). Although the correlation is negative, it represents a positive relationship between mu suppression differences and fantasy, because larger differences in mu suppression are represented by more negative numbers. In contrast, in the context of cotton control, the mu-suppression difference of painful minus non-painful actions was positively associated with participants’ scores on the SPQ’s perspective taking scale (*r* = 0.531, *p* = 0.011; which indicates a negative relationship between mu suppression differences and personal distress; see Figure 7B).

#### Odor Detection

Participants detected neither the chemosensory stress stimulus (median detection rate: 1/5, *Z* = 0.05, *p* = 0.963) nor the sport control stimulus more often than expected by chance (median detection rate: 1/5, *Z* = 1.10, *p* = 0.291). No participant detected the target odor (derived from stress or sport sweat) more than three out of five times.

#### Ratings of Chemosensory Stimuli

The stress odor (*M* = 4.86, *SD* = 2.27) was perceived as more intense than the cotton control (*M* = 3.45, *SD* = 2.13; *t*_(21)_ = 2.46, *p* = 0.023; main effect CHEMOSENSORY CONTEXT: *F*_(2,42)_ = 4.17, *p* = 0.022, $\eta_{\text{p}}^{2}$ = 0.166). Furthermore, the stress odor (*M* = 3.95, *SD* = 2.36) was perceived as more unpleasant than the cotton control (*M* = 2.50, *SD* = 2.02; *t*_(21)_ = 2.54, *p* = 0.019; main effect CHEMOSENSORY CONTEXT: *F*_(2,42)_ = 4.32, *p* = 0.020, $\eta_{\text{p}}^{2}$ = 0.171).

#### Painfulness Ratings

Painful actions were rated as more painful (*M* = 79.05, *SD* = 12.35) as compared to non-painful actions (*M* = 13.75, *SD* = 3.41; main effect picture: *F*_(1,21)_ = 706.47, *p* < 0.001, $\eta_{\text{p}}^{2}$ = 0.971). The chemosensory context did not affect the pain ratings (*p*s > 0.500).

### Discussion

The current study is the first to show that social chemosignals modulate mu-/alpha suppression during action perception. When pictures of non-painful actions are observed, suppression of global alpha activity is more pronounced in the context of stress sweat as compared to a neutral chemosensory context (cotton control and sport sweat). The mu suppressive effect of painful pictures (relative to non-painful pictures), in contrast, nearly vanishes in the context of chemosensory stress signals. Thus, chemosensory signals are at least as potent as visual signals in informing about another individual experiencing threat or harm. Indeed, the harm-related information carried by chemosensory stress signals even seems to override the perception of visual non-pain information. In line with the current results, previous research has shown that stress-related chemosignals are able to tune multimodal perception towards the detection of potential threat (de Groot et al., 2014) and that they even diminish the perception of visual social safety cues (Pause et al., 2004). While it has been discussed before that chemical fear and anxiety is contagiously transmitted from sender to perceiver (Prehn-Kristensen et al., 2009; de Groot et al., 2012), here it is shown for the first time that chemosensory stress signals also affect higher-order cognitive levels of empathy related to perspective taking (Hoenen et al., 2013, 2015).

Stress related chemosignals affect mu activity at central electrodes (reflecting activation of the mirror neuron system; Arnstein et al., 2011), but also alpha activity at occipital electrode sites (reflecting processes associated with arousal and alertness (e.g. Makeig and Jung, 1995; Klimesch et al., 1998). Consequently, chemosensory stress signals might induce empathic states while also increasing general alertness (e.g., Jung et al., 1997; Oken et al., 2006). This interpretation is in line with the finding that in the context of chemosensory stress signals, ambiguous facial expressions attract additional attentional resources (Rubin et al., 2012) and that cortical processing of chemosensory anxiety signals is faster and requires more neuronal resources than the processing of chemosignals derived from sport sweat (Pause et al., 2010).

A similar, but less pronounced effect was found for sport sweat: non-painful pictures elicited stronger suppression of general alpha activity in the context of sport sweat than in the context of cotton control. Accordingly, information conveyed by sweat derived chemicals *per se* might act as a social signal, recruiting attentional resources. This interpretation is supported by studies showing that even non-emotional human body odor is perceived as significant information, acting as social reward signal in socially open individuals (Lübke et al., 2014), and facilitating automatic imitation in autistic children (Parma et al., 2013).

Participants were unable to differentiate the odor from stress and sport sweat from the odor of unused cotton pads. However, the rating differences between cotton pad control and stress related chemosignals, might have been due to priming effects of subthreshold affective information on evaluative ratings (an effect, repeatedly shown for visual affective stimuli; e.g., Murphy and Zajonc, 1993; Pause et al., 2004). Thus, the effects of the chemosensory stimuli on cognitive empathic involvement are considered to have occurred without conscious mediation. Subthreshold effects of social chemosignals on neuronal and muscular activity have been reported repeatedly (e.g., Pause et al., 2004, 2010; Prehn-Kristensen et al., 2009; de Groot et al., 2014; Lübke et al., 2017). Here, it has been shown for the first time that even higher order cognitive processes are susceptible to subthreshold chemical information. Accordingly, the odor ratings of the chemical stimuli must be interpreted cautiously, as they might reflect accidental odor evaluations not being justified by conscious sensory analyses.

It seems to be counterintuitive that participants who scored higher on the perspective taking scale of the SPQ showed less pronounced suppression during the observation of painful pictures (as compared to non-painful pictures) in stress odor context. However, perspective taking (including the reappraisal of observed and experienced emotions) is a necessary component of self-other discrimination, which in turn might reduce cognitive empathy, because it is not adaptive to always share the emotions of others (de Vignemont and Singer, 2006). Similar correlations between perspective taking and mu activity were reported by Woodruff et al. (2011) and Hoenen et al. (2013).

## General Discussion

The current studies show that the mirror neuron system’s response to observed neutral actions can be altered by auditory and chemosensory context. This is in line with studies showing that the mirror neuron system not only represents observed actions, but also the meaning of the actions (e.g., Cheng et al., 2007; Galati et al., 2008; Perry et al., 2010a; Hoenen et al., 2013, 2015).

In the present study, in order to induce empathy, both, the chemosensory and the auditory stimuli are likely to act automatically, not requiring reasoning about the cause of aroused emotions in others (Asada, 2015). Therefore, the auditory and the chemosensory stimuli are prone to directly induce contagious processes (Preston and de Waal, 2001). The main difference between the auditory and the chemosensory conditions was that the screams were perceived as distinct stimuli, but the chemosensory stress-signals could not be detected as odors. Activity within the 8–13 Hz band above central electrodes is related to somatosensory cortex activity and has repeatedly been shown to reflect top-down processes of cognitive empathy (e.g., perspective taking) rather than stimulus driven emotional contagion (Hoenen et al., 2013, 2015). While auditory context signals could have been used to cognitively evaluate the sum of information of all modalities, the chemosensory context changed the cognitive overall evaluation of the potentially harmful situation without being processed as a distinct stimulus. It is concluded that empathic cognitions can be changed through chemosensorily mediated pre-attentive processes.

In contrast to the auditory context, the chemosensory context elicited a general suppression of alpha activity and not a specific suppression of mu activity, which is probably related to differences of the respective stimulus’ specificity. Whereas pain exclamations transport solely pain information, the chemosensory stress signal is less specific to pain but is related to potential harm in general. Therefore, it is reasonable to assume that chemosensory stress signals prime non-specific cortical arousal, instead of eliciting specific activity within the mirror neuron system.

Furthermore, when presented in the context of social chemosignals the observation of painful relative to non-painful actions does not induce mu-/alpha suppression (no main effect of PICTURE ordinal to the PICTURE by CHEMOSENSORY CONTEXT interaction in Study 2), whereas such effect clearly was evident in either auditory context (Study 1). These effect differences are driven by chemosensory stress-signals being more potent than pain-related screams in enhancing mu suppression, even in association with depictions of non-painful actions. Accordingly, social chemosensory stress-signals are more effective than social auditory cues in eliciting activity of neural systems related to empathy and arousal. In comparison to verbal and para-verbal signals, chemosignals are not prone to cheating intentions of the signal sender and can be processed by the perceiver as purely honest information. Therefore, chemosensory signals might affect the multimodal evaluation of social information more effective than signals of other modalities (de Groot et al., 2014; Pause et al., 2004).

It is a particular strength of the present study to systematically compare multimodal effects of visual and auditory stimuli with multimodal effects of visual and chemosensory stimuli on empathy related brain activity. However, in future studies, other perceptual modality combinations may be considered, being of relevance in darkness or for blind individuals (e.g., a combination of auditory and chemosensory stimuli).

## Author Contributions

MH conceived, planned and carried out the experiments, and analyzed the data under supervison of BP. KL contributed to the data acquisition and data analysis. All authors contributed to the interpretation of the results, provided critical feedback and helped shape the research, analysis and manuscript.

## Conflict of Interest Statement

The authors declare that the research was conducted in the absence of any commercial or financial relationships that could be construed as a potential conflict of interest.
